# The interactive effects of different exercises and *hawthorn* consumption on the pain threshold of TMT-induced Alzheimer male rats

**DOI:** 10.1186/s12576-024-00925-4

**Published:** 2024-07-16

**Authors:** Ensiyeh Almasi, Ali Heidarianpour, Maryam Keshvari

**Affiliations:** 1 Department of Exercise Physiology, Faculty of Sport Sciences, Bu-Ali Sina University, Hamedan, Iran; 2 Department of Physical Education and Sport Sciences, Faculty of Literature and Human Sciences, Lorestan University, Khoramabad, Iran

**Keywords:** Alzheimer's disease, Pain threshold, Exercise, *Hawthorn*

## Abstract

Exercise increases the pain threshold in healthy people. However, the pain threshold modulation effect of exercise and *hawthorn* is unclear because of its potential benefits in people with persistent pain, including those with Alzheimer's disease. Accordingly, after the induction of Alzheimer's disease by trimethyl chloride, male rats with Alzheimer's disease were subjected to a 12-week training regimen consisting of resistance training, swimming endurance exercises, and combined exercises. In addition, *hawthorn* extract was orally administered to the rats. Then, their pain threshold was evaluated using three Tail-flick, Hot-plate, and Formalin tests. Our results showed that Alzheimer's decreased the pain threshold in all three behavioral tests. Combined exercise with hawthorn consumption had the most statistically significant effect on Alzheimer's male rats' pain threshold in all three experiments. A combination of swimming endurance and resistance exercises with *hawthorn* consumption may modulate hyperalgesia in Alzheimer's rats. Future studies need to determine the effects of these factors on the treatment and/or management of painful conditions.

**Graphical Abstract:**

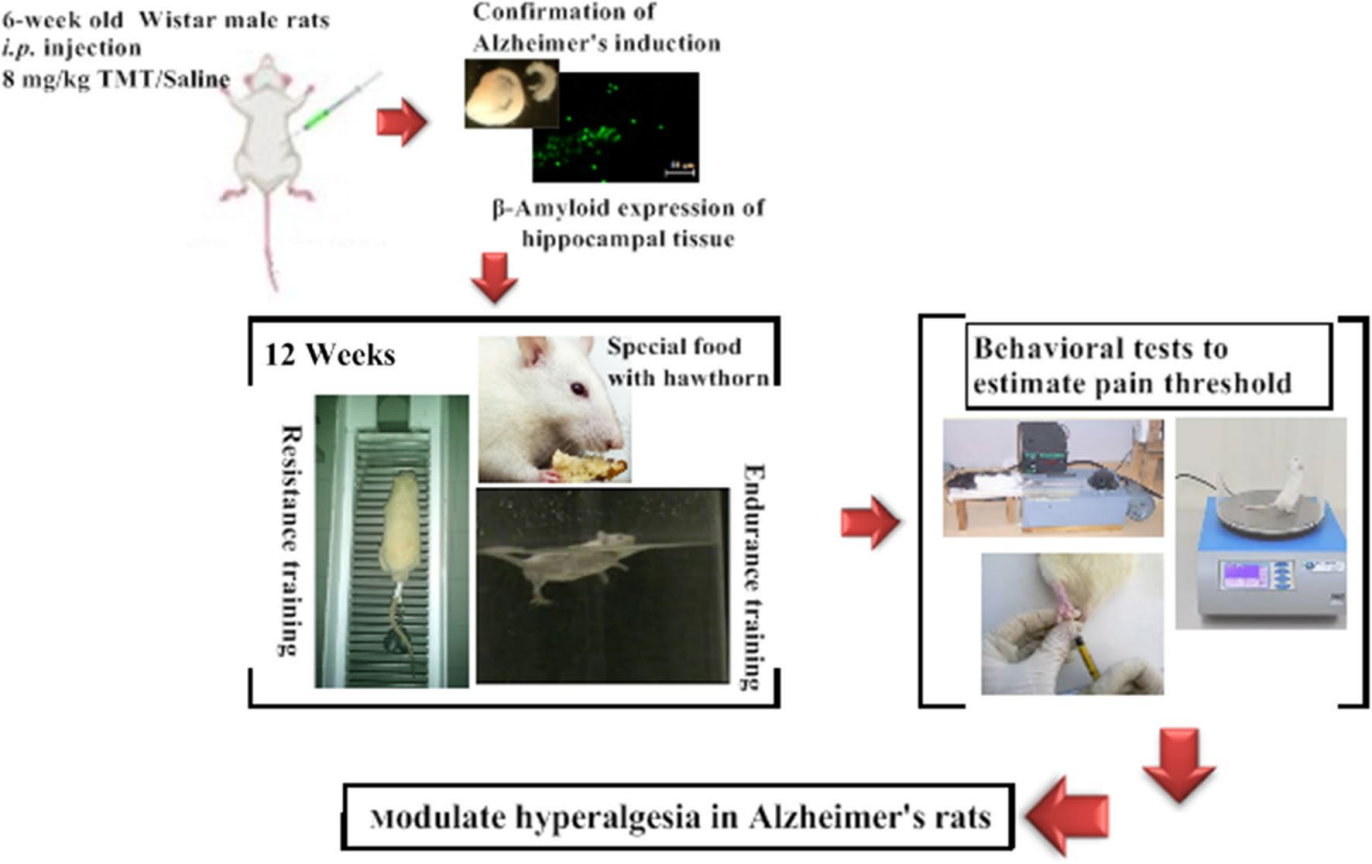

## Introduction

According to the International Association for the Study of Pain (IASP), pain is characterized as an unpleasant sensory and emotional encounter that is linked to or depicted in terms of existing or potential damage to bodily tissues [1]. The nuclei found in the medial thalamus, hypothalamus, cingulate, and insular cortex, which are vital constituents of the intermediate pain system, play a crucial role in processing the emotional–motivational aspect of pain. In general, pain management should primarily be based on its pathophysiological mechanisms as outlined by IASP [2, 3].

Trimethyltin (TMT), a neurotoxin present in the environment, has the potential to induce injury to dopaminergic neurons in the brain [4]. TMT is derived from organotin compounds and is extensively utilized as a heat stabilizer in various industries [5]. Exposure to TMT can result in symmetrical leukoencephalopathy, severe hypokalemia, and neurological symptoms such as headache, amnesia, and weakness [6]. Furthermore, exposure to TMT can lead to neurodegeneration and selective neuronal necrosis [7]. The degeneration caused by TMT exhibits both behavioral and molecular characteristics resembling those observed in Alzheimer's disease (AD) [8]. TMT has been demonstrated to induce alterations in calcium signaling in astrocytes, which in turn leads to mitochondrial depolarization and oxidative stress [8]. In addition, TMT exposure has been found to increase the expression of pro-inflammatory factors and signaling pathway components in astrocyte activation, suggesting a potential role in chronic neuroinflammation [9].

The neuropathological changes resulting from TMT-induced Alzheimer's disease have the potential to impact significant regions involved in the internal pain pathway, such as the internal nuclei of the thalamus, hypothalamus, cingulate cortex, and insula [10]. Conversely, brain regions involved in the lateral path of pain, including the lateral thalamus and primary parietal cortex, generally remain unaffected [11].

A considerable number of AD patients endure severe neuropathic pain [12]. Furthermore, AD induces alterations in the transentorhinal cortex and hippocampus regions [13], resulting in neurological changes within these areas. Remarkably, the cortical areas that make up the peripheral pain network, associated with the sensory-discriminative component (including aspects such as location, sensory attributes, and level of pain), seem to remain largely unaffected by AD [14]. These findings suggest that the assessment of pain, as well as its emotional component (e.g., distress), may be particularly impacted by AD. Moreover, individuals with AD experience a notable decline in cognitive function, making the identification and communication of pain challenging for both sufferers and healthcare practitioners [15].

The accurate evaluation of pain prevalence in the individuals with AD presents inherent challenges, potentially resulting in significant underestimation of the current outbreak rates [15]. However, it is crucial to acknowledge and effectively address pain in individuals with dementia, as failure to recognize its presence is associated with functional deterioration [16], falls [17], and various behavioral and psychological symptoms such as restlessness [18, 19]. Despite the apparent higher prevalence of pain in individuals with dementia compared to their healthy counterparts, there is evidence suggesting that pain in this population is inadequately treated [20, 21].

Pain remains underdiagnosed and undertreated in patients with AD. This underscores the importance of pain management to mitigate behavioral and functional repercussions [22]. An essential aspect of this management involves understanding whether pain sensitivity has changed in individuals with AD, as this knowledge can inform the clinical assessment and treatment of pain in this specific population (4). Notably, one study discovered that the prevalence of pain among individuals with AD ranged from 38 to 75% [23].

Recently, there has been a notable surge in research interest regarding the exploration of non-pharmacological interventions aimed at treating or potentially curing AD. Within this wave of interest, the therapeutic effects of exercise have garnered significant attention [24]. It has been demonstrated through research that exercise has the capacity to alleviate pain [25].

Recent research findings indicate a correlation between exercise and pain threshold. In fact, exercise has the ability to modify the pain threshold. Furthermore, certain scholars argue that there exists a link between the nature and intensity of exercise and relief from pain [26].

In addition, due to the substantial cognitive impairment experienced by individuals with AD, which complicates the identification of pain in this population, the utilization of herbal remedies such as *hawthorn* (Haw) may prove beneficial in ameliorating cognitive impairment and facilitating the timely detection of pain [27, 28]. Modern pharmacological research has revealed the potential of Haw as a therapeutic approach for the treatment of AD, with its effects encompassing the enhancement of neurobehavior and improvement of cognitive impairment [28].

Flavonoid-enriched foods, like Haw [29], can potentially serve as a novel therapeutic strategy for neuropathic pain in preclinical models, though there is currently no exploration of clinical evidence in human beings [30]. In this review, we will discuss the evidence supporting the analgesic and anti-inflammatory properties of flavonoids [31]. Flavonoids have been reported to alleviate neuropathic pain in mouse models when used for the management of neuropathic pain conditions [32]. Although extensive research has been conducted on the relationship between exercise and learning memory, limited insight is available related to the impact of exercise activity combined with Haw consumption on AD-induced behavioral changes, including pain threshold. Given that the perception of pain is crucial for survival, it is imperative to evaluate it even in the absence of reliable subjective reports, particularly in individuals with severe cognitive impairment. Neuropathological alterations occurring in individuals with dementia are considered to be responsible for the changes in pain perception. While these changes may be experienced across various types of dementia, the majority of the clinical and experimental studies investigating pain assessment or treatment in dementia have focused on patients diagnosed with AD. According to a widely accepted theory, the degeneration of AD primarily affects the affective–motivational aspect of pain (medial pathway) more than the sensory discriminative dimension (lateral pathway). In addition, the cognitive impairment commonly observed in AD, characterized by memory deficits and reasoning difficulties, may impact the individual's ability to assess and describe a painful experience. Based on this clinical theoretical framework, it is proposed that AD patients may exhibit an unaltered pain threshold and an increased tolerance for painful stimuli, which warrants further investigation [33, 34].

While extensive research has been conducted on the relationship between exercise and memory and learning, there is a limited information available regarding the impact of exercise associated with Haw consumption on behavioral changes induced by AD, including the pain threshold. Therefore, the present study was set up to investigate the effect of Haw consumption and three models of endurance, resistance and combined exercises on the pain threshold of AD rats induced with TMT.

## Materials and methods

### Animals and experimental groups

**Fig. 1:**
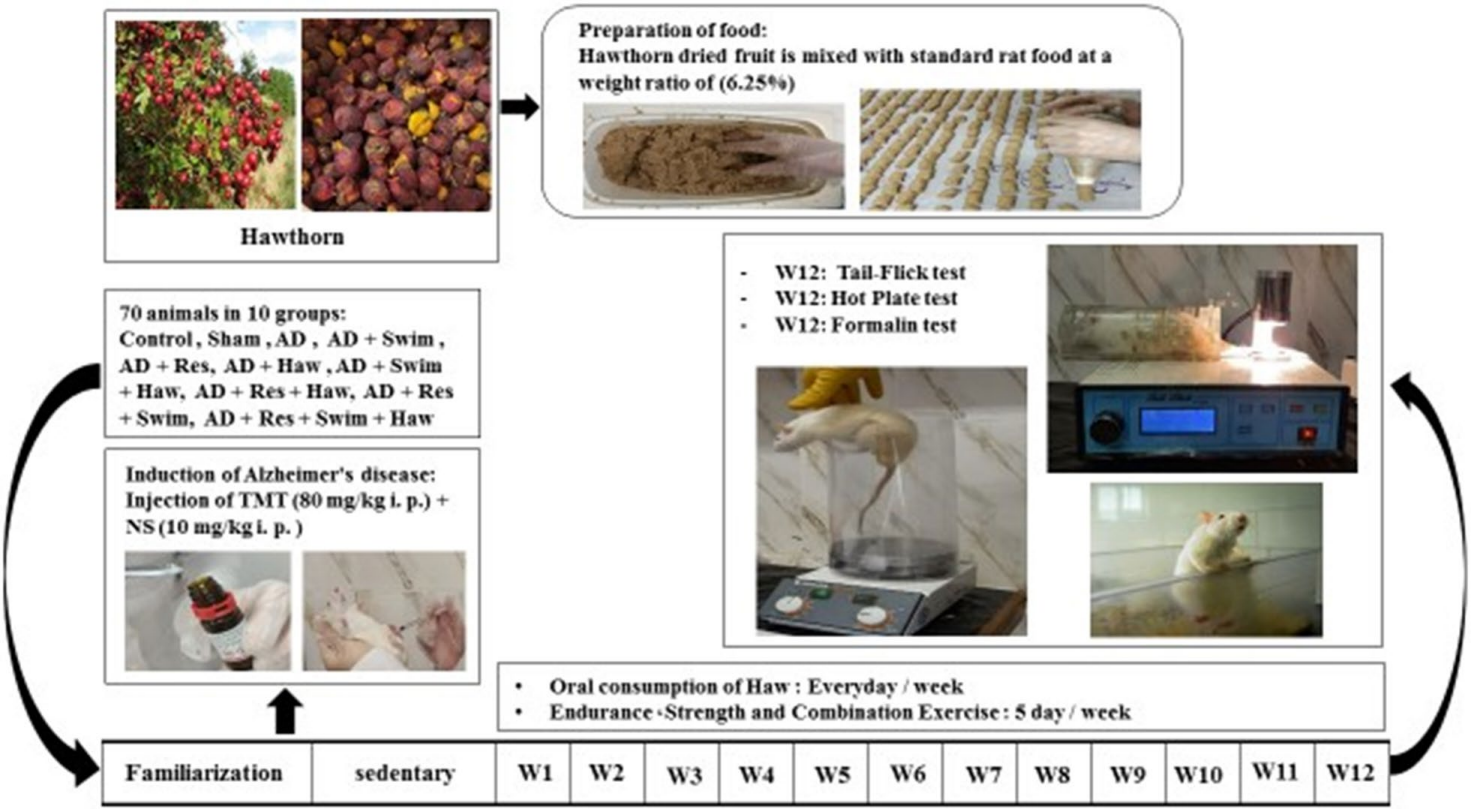
Experimental design

In this experimental investigation, a total of 80 Wistar male rats (6 weeks, 200 ± 20 g), were procured from the Animal Laboratory of Bu Ali Sina University. Throughout the study period, the rats were kept in a controlled environment at a temperature of 22 ± 2 °C and a humidity level of 50% ± 5% with a balanced exposure of 12 h of light/darkness. In addition, all the rats had free access to food and water. After transferring the rats to the laboratory and adapting them to the new environment, as well as familiarizing them with exercise protocols, the rats were randomly divided into ten equal groups. These groups encompassed the following categorizations: Healthy Control (Control), Alzheimer's Control (AD), Sham (Shams), Alzheimer + Swimming exercise (Swim), Alzheimer + *Hawthorn* (Haw), Alzheimer + Swimming exercise + *Hawthorn* (Swim + Haw), Alzheimer + Resistance exercise (Res), Alzheimer + Resistance exercise + *Hawthorn* (Res + Haw), Alzheimer + Combined exercise (Res + Swim), and Alzheimer + Combined exercise + *Hawthorn* (Res + Swim + Haw). This study was carried out in accordance with the code of ethics registered in Bu Ali Sina University (IR BASU.REC.1400.007(. The graphic abstract of the research protocol testing steps is given in Fig. 1.

### Induction of Alzheimer's disease with TMT

Trimethyltin chloride (TMT) is an organotin compound [35]. Administration of TMT induces cognitive deficits in experimental models known as severe hippocampal neuronal damage [36]. The first aim of TMT is the hippocampus, where it has poisonous effects on pyramidal neurons. Structural damage begins 2–3 days after TMT injection [37]. 1 week after their adaptation to the environment and exercise training, the process of developing AD in rats began with the administration of TMT. To prepare TMT and inject it intraperitoneally into rats, 8 mg/kg of this substance was dissolved in 1 ml of normal saline solution [10, 38, 39].

### Confirmation of Alzheimer's induction

Ten days after TMT drug injection, the animals showed behavioral changes such as muscle tremors, body temperature increase, nausea, seizures, tail twisting, aggressive behavior, self-harm, impaired working memory, and hyperactivity. Based on the studies, the emergence of these clinical symptoms confirms the induction of AD [10, 38, 39]. After confirming the induction of AD by evaluating the behavioral symptoms, the hippocampal tissue of six rats was randomly removed for the final confirmation of the induction of AD by examining the expression of beta-amyloid protein. Then, the hippocampal tissue was fixed in 10% formalin. In the next step, according to the tissue preparation steps for staining, they were dehydrated in different percentages of alcohol and clarified by xylol. After molding the samples with paraffin, 5 µm thick sections were taken from all the molds using a rotary microtome [40]. After dehydration, slides were incubated in 50% formaldehyde and 2X sodium citrate buffer at 65 °C for 2 h, and then they were washed twice in 100 µm sodium borate buffer (pH = 8.5). To denature the DNA, the samples were incubated with 2 normal HCL at 37 °C and then washed with phosphate buffered saline (PBS). The samples were blocked with Triton- × 100 (0.3%) and goat serum (10%) in PBS for 30 min [40]. Finally, the samples were incubated with primary antibodies of β-Amyloid Polyclonal (E-AB-70168) for 1 night at a temperature of 2–8°. In the next step, the slides were incubated with secondary antibody (ab6785) connected to fluorescent FITC for 90 min at 37°. Then, to stain the nuclei, the slides were incubated with 6-4-diamidino-2-phenylindole (DAPI) for 15 min. After lameness, the slides were examined by a fluorescent microscope (Olympus BX50). Finally, using Image J software version 1.5, proteins were measured by checking the density of green pixels in the captured images.

### Preparation of special food with *hawthorn*

The verification of Haw fruits was conducted by the herbarium at Bu Ali Sina University subsequent to their identification and collection from the Zagros Mountain region situated in western Iran. An initial step in the creation of a specialized pellet containing Haw involved subjecting the Haw fruits to a week-long drying process within a covered environment. Subsequently, the dry fruits of Haw were ground with a special spice, and the resultant powder was amalgamated with a 6.25% weight proportion of powdered rat food, produced by Behparvar Animal Feed Company, along with water to form a cohesive paste. The resulting mixture was funneled and segmented into pellets suitable for consumption by rats. Following this, the pellets underwent a drying phase within an enclosed environment [41]. The rats in the sham, control, and other groups that did not receive Haw intervention consumed normal food from the Behparvar Animal Feed Company. The rats in all groups had free access to food and water without any restrictions.

The compositions of Haw extracts were determined by HPLC. The calibration range of each compound was as follows: catechin (0.16–36.6 μg), chlorogenic acid (0.75–9.80 μg), epigallocatechin gallate (0.21–32.76 μg), rutin (0.62–9.70 μg), vitexin (0.37–5.95 mg), isocercetin (0.66–16.40 μg), quercetin (0.11–11.40 μg), methoxykaempferol-hexoside (0.87–5.74 μg), apigenin (0.18–8.0 μg), gallocatechin gallate (0.14–4.87 μg), vitexin-2″-O-rhamnoside (0.84–19.45 μg), isoquercitrin (0.54–6.98 μg), hyperoside (0.43–8.23 μg), and resveratrol (0.85–25.8 μg).

### Exercise training protocol

#### Endurance swimming exercise protocol

The swimming exercise protocol was performed for 12 weeks and 5 sessions per week. Endurance swimming exercise was performed in a special swimming pool made of glass (80 × 60 × 100 cm) with a water temperature of 25–30 °C. During the exercise, a wave pump was used to prevent the rats from floating. The sequence of exercise days was: 3 days of exercise, 1 day of rest, then 2 days of exercise, and 1 day of rest. The swimming duration ranged from 5 to 15 min in week 1, 20 min in week 2, 30 min in week 3, 45 min in week 4, 50 min in week 5, and 60 min from weeks 6 to 12. Concerning the frequency of the training sessions, throughout the first 9 weeks, the training consisted of one session per day, progressing to two times per day with a 4-h gap in the 10th week. Subsequently, it was increased to three times daily with a 3-h hiatus in the 11th week, and then, it reached four times a day with a 2-h break in the 12th week (Table 1) [42].

**Table 1:** Swimming protocol

Week No.	Swimming duration	Recovery between training sessions
1	5–15 min	24 h
2	20 min	24 h
3	30 min	24 h
4	40 min	24 h
5	50 min	24 h
6–9	60 min	24 h
10–12	60 min	12 h

#### Resistance exercise protocol

Resistance exercise was performed for 12 weeks, 5 sessions per week. This protocol was in the form of climbing a ladder with weights attached to the rats' tails. The ladder used was 1 m long with 26 steps and an angle of 85° to the ground. The initial phase of the resistance training protocol involved familiarizing the rats with climbing a ladder while progressively increasing the load attached to their tails based on a percentage of their body weight. Subsequently, the load was set at 30% in the second week, 70–90% in the third-to-fifth weeks, 100–110% in the sixth-to-eighth weeks, 120–130% in the ninth and tenth weeks, and 140–150% in the 11th and 12th weeks. Each training session comprised 3 sets of 4 repetitions, with rest periods of 30–60 s between each repetition and 120–150 s between each set (Table 2) [43].

**Table 2:** Resistance protocol

Week No.	Percentage of body weight	The interval between each repetition (s)	The interval between each set (s)
1–2	50–60	30	120
3–4	70–80	30	120
5–6	90–100	30	120
7–8	105–110	30	120
9–10	120–130	50	140
11–12	140–150	60	150

#### Combined exercises

The combined protocol was practiced 2 days of resistance exercise per week, and 3 days of endurance swimming exercise per week according to the intensity and duration of high exercise protocols.

### Tail-flick test

After the completion of exercise and dietary interventions, a period of 48 h transpired before we initiated the evaluation of pain thresholds. The assessment tests employed were the teal flick test, followed by the Hot-plate test, and finally the Formalin test. These tests were conducted at intervals of 24 h. To assess anti-nociception, the Tail-flick test was employed [44]. The intensity of the beam was adjusted to establish a mean control reaction time of 4–6 s. In order to prevent harm to the tail, a cutoff time of 15 s was implemented. In this manner, we were able to identify any potential and subtle variations that were likely to occur in the pain related to thermal stimuli. The baseline latency for the Tail-flick test was determined by conducting three measurements for each rat and then calculating the mean. An increase in the latency of the control Tail-flick test induced by the experiment served as an indicator of antinociceptive effect, while a decrease in the latency of the Tail-flick test signified a hyperalgesic effect [45].

### Hot-plate test

The rats were subjected to a Hot-plate with a temperature of 55 ± 0.2 ◦C. During this period, their actions of licking the hind paws or attempting to escape from the glass enclosure were observed and recorded as the latency period. To prevent any harm to the rats, a maximum duration of 60 s was set as the cutoff time, thus avoiding any potential damage to their paws [46].

### Formalin test

The Formalin test was conducted in accordance with the procedure outlined by Dubuisson and Dennis [47]. A volume of 10 µ‏l of a 5% solution of formalin in saline was administered into the dorsal surface of the left hind paw using a tuberculin syringe. Subsequently, each animal was placed within an observation chamber and subjected to continuous monitoring for 1 h. The intensity of pain responses was duly documented utilizing a standardized scale: (0) rats demonstrated normal ambulatory behavior or maintained a stable stance on the injected claw; (1) the injected claw was partially raised; (2) the injected claw was completely lifted off the ground; (3) the rats displayed licking, chewing or shaking of the injected claw. This particular scoring methodology permits the classification of responses, thereby allowing for a more precise assessment of analgesia levels, in contrast to the conventional approach, which solely accounts for the duration of time spent on licking the injected claw. The analgesic effect was evaluated in two distinct phases, namely the early acute phase and the subsequent late or tonic phase (0–5 and 15–60 min after formalin injection, respectively).

### Statistical analyses

Descriptive and inferential statistics were employed following to the completion of data collection. Levene’s test was utilized in assessing the equality of variances, while the Shapiro–Wilk test was employed to ascertain normal data distribution. The two-way ANOVA test was performed to explore the Alzheimer* *hawthorn* interaction in the behavioral test. All findings are reported as Mean ± SD, with statistical significance defined as *P* < 0.05. The statistical analysis was performed using SPSS software version 22.

## Results

### Induction of Alzheimer's disease with TMT

**Fig. 2:**
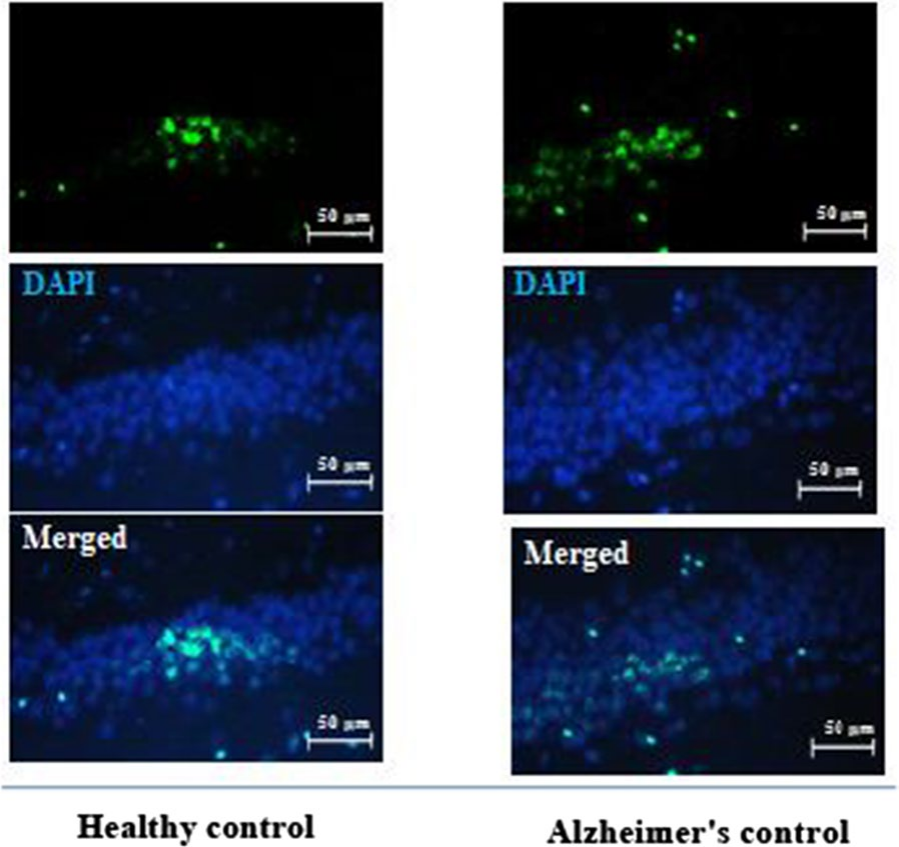
β-Amyloid expression level of hippocampal tissue in Alzheimer's control rats and healthy controls. β-Amyloid expressed in the CA1 region of the hippocampus is highlighted in green in the immunofluorescent images

The results of investigating the expression of β-Amyloid plaques in the hippocampus of rats after 10 days of TMT injection showed a significant difference (*P* = 0.001, *t* = − 34.73) in the average expression of β-Amyloid plaques between the control (% 9.42 ± 0.61) and Alzheimer's groups (% 43.80 ± 1.60) (Fig. 2).

### Tail-flick test

In order to investigate the effect of hawthorn*exercise on pain threshold variable, Tail-flick Two-way analysis of variance was used. The result of this test showed a significant interaction between Haw and exercise on the pain threshold of Alzheimer's rats (*F* = 33.892, *P* = 0.001, *η*^2^ = 0.339).

The measurement of pain threshold revealed that TMT-induced Alzheimer's significantly reduced the pain threshold in AD rats compared to healthy rats (*P* = 0.001). 12 weeks of exercise and Haw increased the pain threshold in the Swim (*P* = 0.048), Res + Haw (*P* = 0.001), Swim + Haw (*P* = 0.001), Res + Swim (*P* = 0.003), and Res + Swim + Haw (*P* = 0.001) groups compared to the AD group.

**Fig. 3:**
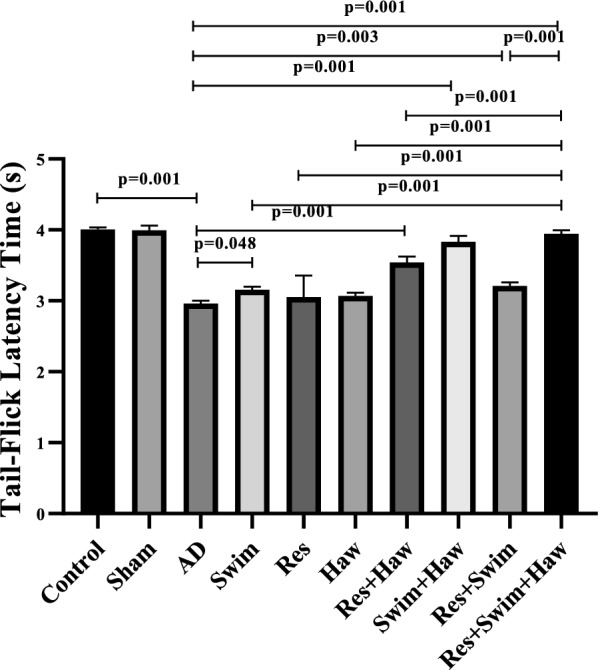
Latency in tail flick test

Among the intervention groups, the pain threshold was higher in the Res + Swim + Haw combined group, which had a total of endurance and resistance exercise interventions with Haw consumption, compared to other groups except Swim + Haw (*P* = 0.001) (Fig. 3).

### Hot-plate test

The interaction of hawthorn*exercise was significant in the pain threshold of Alzheimer's rats performing the Hot-plate test (*F* = 10.355, *P* = 0.002, *η*2 = 0.136). The measurement of pain threshold by Hot-plate test showed that TMT-induced Alzheimer's significantly reduced the pain threshold in AD rats compared to healthy rats (*P* = 0.001).

**Fig. 4:**
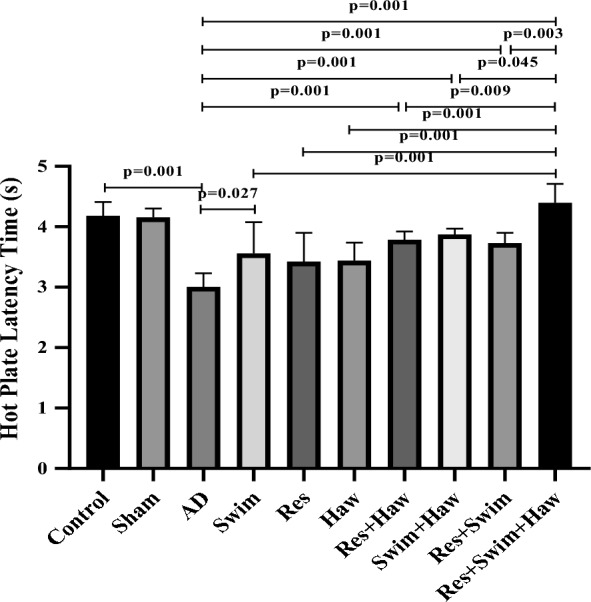
Latency in hot plate test

Thus, 12 weeks of exercise and Haw consumption increased the pain threshold in the Swim (*P* = 0.027), Res + Haw (*P* = 0.001), Swim + Haw (*P* = 0.001), Res + Swim (*P* = 0.001), and Res + Swim + Haw (*P* = 0.001) groups compared to the AD group. One of the interesting results of the Hot-plate test was that the Res + Swim + Haw group had the highest pain threshold compared to other intervention groups (*P* < 0.05) (Fig. 4).

### Formalin test

The interaction of exercise* *hawthorn* on the pain threshold was significant in the early (*F* = 41.463, *P* = 0.001, *η*^2^ = 0.386) and final (*F* = 37.247, *P* = 0.001, *η*^2^ = 0.361) phases of the Formalin test.

#### Early phase (0–5 min)

Measurements showed that TMT-induced Alzheimer's caused a significant increase in pain response time to formalin injection, so the pain response time in the first seconds of formalin injection (0–5 min) in Alzheimer's rats in the AD group increased significantly compared to the rats in the healthy group (*P* = 0.001). Twelve weeks of exercise training and consuming Haw alone and together decreased the response time to pain in Res + Haw (*P* = 0.001), Swim + Haw (*P* = 0.001), Res + Swim (*P* = 0.001), and Res + Swim + Haw (*P* = 0.001) compared to AD group, which indicates an increase in the pain threshold in these groups. One of the interesting results was that the Res + Swim + Haw group had the fastest pain response time compared to other groups (*P* < 0.01).

#### Late phase (15–60 min)

**Fig. 5:**
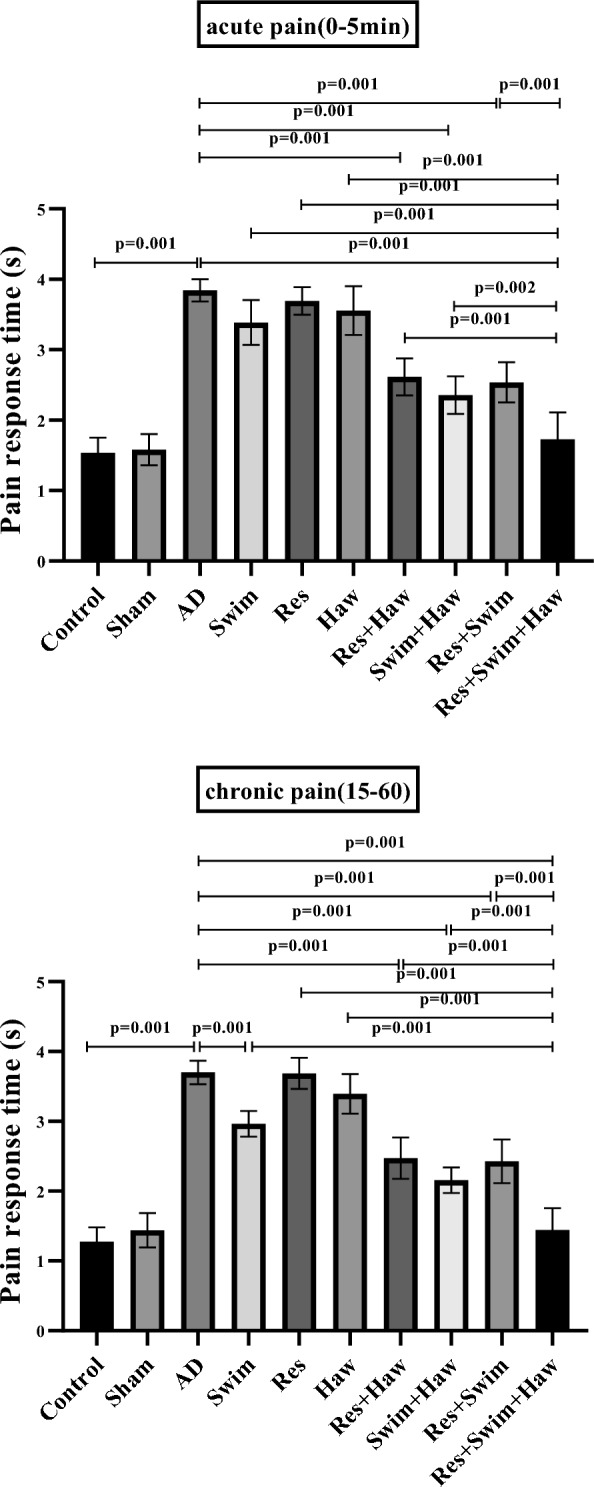
Times of pain response to injected formalin in the formalin test

The measurements revealed that TMT-induced Alzheimer's caused a significant increase in the pain response time to injected formalin in secondary seconds (15–60 min) of Alzheimer's rats in the AD group compared to the rats in the healthy group (*P* = 0.001). Twelve weeks of exercise training and consuming Haw alone and together, reduced the response time to pain in the Swim, Res + Haw, Swim + Haw, Res + Swim, and Res + Swim + Haw groups compared to the AD group (*P* = 0.001). In this phase of the Formalin test, the rats of the Res + Swim + Haw group displayed the shortest pain response time compared to other groups (Fig. 5).

## Discussion

In this research, the evaluation of the pain threshold of TMT-induced AD male rats in all three tests including Hot-plate, Tail-flick, and Formalin showed that AD significantly reduces the pain threshold. Individuals with AD exhibit heightened pain sensitivity and endure greater pain compared to healthy individuals [48], as well as experience severe neuropathic pain [49]. The neuropathological alterations caused by AD potentially affect crucial regions involved in the medial pain pathway [49].

Pain in people with AD is accompanied by a decreased ability to communicate pain and often misdiagnosis and inappropriate pain management. The experience of pain is a key factor in the challenge of caring for people suffering from AD and is often associated with age-related medical conditions. Despite the availability of pain treatment options, pain in AD is often difficult to assess and treat, with pain often having a negative impact on quality of life. Incorrect and inappropriate prescription in this group of patients leads to reduced mobility, muscle weakness and falls, which has a major detrimental effect on their quality of life and plays an important role in the occurrence of neuropsychological symptoms such as aggression and mood disorders [50]

Individuals with early AD have been found to have a lower perception of pain intensity compared to cognitively healthy elderly individuals [48] implying that during the early stages of the disease, the comprehension and documentation of pain may already be disrupted. These discoveries have prompted the inquiry as to whether AD results in a modification in the perception of pain owing to altered pain processing linked with neurodegenerative changes. Nevertheless, this matter has only been explored in a restricted number of experimental investigations, and so far, no consistent findings have been observed. For instance, Benedetti et al. did not detect any disparities in pain threshold between AD patients and healthy control subjects when employing electric and ischemic stimuli [51]. However, they did note an augmented tolerance to pain in AD patients compared to healthy controls [52].

On the other hand, Cole et al. used pressure pain stimuli and observed an increased threshold in the perception of noticeable pain [14]. These contradictory findings are attributable to methodological differences in pain induction and assessment as well as the severity of AD. In addition, it remains unclear whether the methods used are suitable for patients with AD. Due to their short-term memory disorder and difficulties in perceiving simple instructions, patients with AD may not be able to reliably use certain assessment methods. Therefore, some of the discrepancies found in the literature regarding pain understanding in AD patients may be a result of the inappropriate use of assessment methods that are not reliable for this population [53].

According to certain theoretical frameworks, it necessitates further inquiry to ascertain the presence of an elevated pain threshold and an increased tolerance for painful stimuli in individuals afflicted by AD [33, 34]. In the course of this investigation, the conducted analyses demonstrated a noteworthy reduction in pain threshold among rats with AD. However, the absence of a pharmacologically efficacious medication for the treatment of this ailment is evident. Consequently, botanical organisms possess the potential to serve as intriguing reservoirs for the development of anti-Alzheimer's drugs [54]. The presence of antioxidant compounds assumes a pivotal role in the preservation of optimal well-being. By way of illustration, the accumulation of compounds such as phenolic acid, polyphenols, and flavonoids facilitates the neutralization of peroxide and hydro-peroxide radicals, thereby impeding oxidative mechanisms that give rise to ailments characterized by heightened susceptibility [55].

The findings from epidemiological investigations and animal experiments indicate that diets abundant in flavonoids can yield a beneficial impact on cerebral functioning and decrease the occurrence of neurodegenerative disorders, such as Alzheimer's and Parkinson's [29, 56, 57]. Moreover, it has been documented that flavonoids can alleviate neuropathic pain in rat models [58]. Haw, which comprises flavonoid compounds including Quercetin, chlorogenic acid, and anthocyanins, has been demonstrated to exhibit anti-inflammatory, neuroprotective, and analgesic effects [59–62], thus signifying its potential for reducing neuropathic pain. Based on the outcomes of our investigation, we have ascertained that the consumption of Haw raises the threshold for pain in rats with Alzheimer's. This augmentation in pain threshold observed among the Alzheimer's rats that consumed Haw is attributable to the ability of flavonoids present in Haw to diminish neuropathic pain, which corroborates the findings of the aforementioned studies. Furthermore, Haw is replete with antioxidants, and contemporary pharmacological research has indicated that Haw serves as a therapeutic approach for treating AD and thwarts the accumulation of β-Amyloid in the brain [28].

Studies show that physical activity has the capability to modify the perception of pain [63, 64], and elevate the threshold at which pain is experienced [65]. In addition, it has been demonstrated that exercise can activate certain physiological systems that are also stimulated by opioids such as morphine [66]. This phenomenon is commonly referred to as exercise-induced hypoalgesia (EIH) and is closely associated with the effectiveness of endogenous pain-inhibitory pathways. Currently, EIH has been observed consistently in various types of exercise including aerobic exercise, dynamic circuit resistance exercise, and isometric exercise. These exercises have frequently been found to result in heightened pain thresholds and diminished pain ratings. However, the fundamental neural mechanisms underlying EIH have not been fully comprehended. The evidence from animal studies suggest that the activation of the motor cortex during exercise leads to sustained inhibition of the nociceptive system. Specifically, it has been observed that the activation of the motor cortex in primates can reduce the excitability of the spinothalamic neurons. Nevertheless, the exact role of exercise in modulating pain is not entirely clear. A thorough investigation into the mechanisms by which exercise influences pain control is imperative. Accordingly, the present study aimed to examine the impact of endurance swimming exercises and resistance exercises, both separately and in combination, on the modulation of pain perception [67].

Regular physical activity enhances cerebral blood flow, and aquatic exercise promotes greater cerebral blood flow when compared to land-based exercise of similar intensity [68]. The investigation of three distinct pain models, namely the Hot-plate, Tail-flick, and Late Phase Formalin tests, demonstrated that Alzheimer's rats engaged in swimming exercise exhibited a higher pain threshold than the ones performing resistance exercise, in line with the findings of Pugh et al. This discrepancy can be attributed to the heightened cerebral blood flow observed during water-based exercise in contrast to land-based exercise. The outcomes of this study revealed that the simultaneous execution of endurance swimming exercises and resistance exercises elicited a more substantial elevation in pain threshold compared to the separate performance of each exercise.

This study employed three empirical pain models to assess the analgesic properties of exercise and Haw. The selection of these methodologies aimed to explore both centrally and peripherally mediated effects. The Tail-flick and Hot-plate tests primarily assessed central activity, while the Formalin test provided insights into both central and peripheral effects. Pain information undergoes processing and integration at various levels within the central nervous system, including environmental, spinal, and supraspinal levels. The Tail-flick test represents a spinally integrated nociceptive reflex, whereas the Hot-plate test encompasses a more intricate response that integrates supraspinally. The combined utilization of exercise and Haw consumption during the Tail-flick and Hot-plate tests yielded the most significant impact on pain threshold. This suggests the analgesic effects of exercise and Haw at both spinal and supraspinal levels, as assessed by the Tail-flick and Hot-plate tests.

In the Formalin test, a biphasic pain response consisting of early and late phases was observed. Pharmacological agents that predominantly target the central nervous system inhibit both phases, whereas peripheral drugs primarily inhibit the terminal phase. The concurrent implementation of exercise and Haw consumption during the Formalin test resulted in the most substantial alterations in pain response time during both phases. This underscores the simultaneous impact of combined exercise and Haw on the central nervous system. According to the study, the simultaneous implementation of exercise and herbal interventions lead to the greatest increase in pain threshold.

The constraints of this research in investigating the impact of exercise and Haw on the pain threshold of male Alzheimer's Wistar rats included challenges in selecting animal models and determining the sample size. Animal models for Alzheimer's disease may not completely mirror the human condition, impacting the applicability of results to clinical trials [69]. Furthermore, discrepancies existed in the development of Alzheimer's disease in male and female field rats, complicating gender comparisons in Alzheimer's disease. These constraints underscore the significance of a thorough assessment of animal data prior to advancing to clinical trials, as well as the necessity for strong experimental design and reporting criteria in animal research to enhance their credibility and replicability.

## Conclusion

The results of this study demonstrate that the combination of exercise and Haw consumption yields the most significant impact on pain threshold. No research has examined the joint influence of these two factors on Alzheimer's disease or pain. However, the rationale behind the heightened effectiveness of these two factors in conjunction can be explicated by the association between AD and the heightened production of oxidative stress. This surge in oxidative stress serves as one of the catalysts for neuropathy and the subsequent decrease in pain threshold. Conversely, Haw, acting as an antioxidant, counteracts the repercussions of oxidative stress. Moreover, the presence of flavonoids in Haw reduces central sensitivity to pain, thereby elevating the pain threshold. When these effects of Haw are combined with the antioxidant and opioid impacts of exercise, they yield the most profound influence on pain threshold.

## Data Availability

The data underlying this article will be shared on reasonable request to the corresponding author.

## Abbreviations

AD: Alzheimer's disease
IASP: International Association for the Study of Pain
HAW: *Hawthorn*
Res: Resistance Exercise
Swim: Endurance swimming exercise
TMT: Trimethyltin chloride
CGA: Chlorogenic acid
EIH: Exercise-induced hypoalgesia
